# ﻿On the specific status of *Scelimenaspicupennis* and a new record of *S.discalis* from China with mitochondrial genome characterization (Orthoptera, Tetrigidae)

**DOI:** 10.3897/zookeys.1185.110148

**Published:** 2023-11-28

**Authors:** Ying-Can Qin, Jing Liu, Xiao-Dong Li, Ya-Zhen Chen, Wei-An Deng

**Affiliations:** 1 Key Laboratory of Ecology of Rare and Endangered Species and Environmental Protection (Guangxi Normal University), Ministry of Education, Guilin, Guangxi 541006, China Guangxi Normal University Guilin China; 2 School of Chemistry and Bioengineering, Hechi University, Yizhou, Guangxi 546300, China Hechi University Yizhou China; 3 Guangxi Key Laboratory of Rare and Endangered Animal Ecology, Guangxi Normal University, Guilin, Guangxi 541006, China Guangxi Normal University Guilin China; 4 College of Life Sciences, Guangxi Normal University, Guilin, Guangxi 541004, China Hechi University Yizhou China

**Keywords:** Mitochondrial, morphological systematics, Pygmy grasshopper, phylogenomics, Scelimenini, taxonomy, Tetrigoidea

## Abstract

The genus *Scelimena* Serville (Orthoptera: Tetrigidae) from China is reviewed. One species, *Scelimenaspicupennis* Zheng & Ou, 2003 (China: Yunnan) is redescribed, and a new record of *Scelimenadiscalis* (Hancock, 1915) from China is given. An annotated identification key for Chinese species of the genus *Scelimena* is provided. Mitochondrial genes of *S.spicupennis* and *S.discalis* were sequenced and annotated. The sizes of the two sequenced mitogenomes are 17,552 bp (*S.discalis*), and 16,069 bp (*S.spicupennis*), respectively. All of the PCGs started with the typical ATN (ATT, ATC or ATG) or TTG codon and most ended with complete TAA or TAG codon, with the exception of the ND5 gene, which terminated with an incomplete T. The mitochondrial genomes for these two recorded species are provided, and the constructed phylogenetic tree supports their morphological taxonomic classification. The topology of the phylogenetic tree showed that three species of *Scelimena* were clustered into one branch and formed a monophyletic and a holophyletic group.

## ﻿Introduction

The genus *Scelimena* Serville belongs to the subfamily Scelimeninae, tribe Scelimenini (Orthoptera: Tetrigidae) with the type species *Scelimenaproducta* Serville, date. To date, the genus includes 23 known species distributed mainly in the Oriental biogeographical realm (Lao et al. 2022; Muhammad et al. 2023), i.e., in India, Bangladesh, Myanmar, Thailand, Vietnam, Laos, PR China, Peninsular Malaysia, Sumatra, Borneo, Palawan Isl., Philippines, Java, East Sumba, Sulawesi, Flores, and New Guinea with adjacent small islands. These 23 known species include six species groups: *bellula* Storozhenko & Dawwrueng, 2015, *discalis* Hancock, 1915, *hexodon* Haan, 1843, *novaeguineae* Bolívar, 1898, *producta* Serville, 1838, and *spiculata* Stål, 1877.

PR China is known to be inhabited by at least six *Scelimena* species, of which five have been assigned to a species group. These are (1) *S.guangxiensis* Zheng & Jiang, 1994 (*S.bellula* species group), (2) *S.melli* Günther, 1938a (*S.bellula* species group), (3) *S.nitidogranulosa* Günther, 1938b (*S.bellula* species group), (4) *S.songkrana* Zha & Wen, 2017 (*S.discalis* species group), (5) *S.pyrroma* Lao, Kasalo, Gao, Deng & Skejo, 2022 (*S.producta* species group), and (6) *S.spicupennis* Zheng & Ou, 2003. The original description of *S.spicupennis* was simple and inaccurate and because no further work was done on this species, it does not have a species group assignment.

Urgent systematic studies were therefore required to address the complex phylogeny of Scelimeninae. Emerging advances in sequencing technology and burgeoning strides in molecular biology have complemented the escalation of research interests in the study of the mitogenome of Scelimeninae. Earlier studies by Chen et al. (2018) employed a combination of COI, 16S rRNA, and 18S rRNA genes data sets to delineate phylogenetic relationships among Scelimeninae genera. However, their conclusion that Scelimeninae may be monophyletic group faced opposition from Adžić et al. (2020) who claimed they are not. Further more, Li et al. (2021) sequenced eight nearly complete mitogenomes of Scelimeninae and constructed phylogenetic trees, converging to the same disputed conclusion as Adžić et al. (2020). Nevertheless, they also emphasized the necessity for more extensive mitogenomic taxon sampling to unveil the intricate phylogenetic relations within Scelimeninae.

The current study aims to investigate two *Scelimena* species from China:

(1) *Scelimenaspicupennis* Zheng & Ou, 2003, for which almost no data exist.

(2) *Scelimenadiscalis* (Hancock, 1915), for which this is the first record from China.

Detailed redescriptions, illustrations, and comparative analyses with closely related species are proffered for these species. Further, the study provides mitochondrial genomes for the identified species and exploits 13 protein-coding genes to construct phylogenetic trees of the related species via Bayesian inference (BI) and maximum likelihood (ML) methodologies.

## ﻿Materials and methods

Taxonomy follows Orthoptera Species File [OSF] (Cigliano et al. 2022), a database of Orthoptera taxonomy. Nomenclature is following the International Code of the Zoological Nomenclature (ICZN 1999). Morphological terminology and landmark-based measurement method followed those used by Zheng (2005), Deng et al. (2007), Tumbrinck (2014), Muhammad et al. (2018) and Tan and Artchawakom (2015). Measurements are given in millimetres (mm). The specimens examined in this study, including holotype and paratypes, have been deposited in the College of Life Sciences, Guangxi Normal University, Guilin, China (CLSGNU).

Terminology of the pronotal projections follows Pushkar’s system (Skejo and Bertner 2017; Storozhenko and Pushkar 2017; Skejo et al. 2022), and most important are the following:

**FM** frontomedial projection;

**FL1** first frontolateral projections;

**FL2** second frontolateral projections;

**PM** promedial projection;

**MM1** first metamedial projection;

**MM2** second metamedial projection;

**MM3** third metamedial projection;

**ML (ML1)** first metalateral projections;

**ML2** second metalateral projections;

**ML3** third metalateral projections;

**MML1** first metamediolateral projections;

**MML2** second metamediolateral projections;

**MML3** third metamediolateral projections;

**VL** ventrolateral projections.

Total genomic DNA was extracted from muscle tissues of the hind femur of each sample using the TIANamp Genomic DNA Kit (TIANGEN) and sent to Shanghai Yaen Biotechnology Co., Ltd. for high-throughput sequencing. Using the mitochondrial sequence of *Scelimenamelli* (GenBank accession number: MW722938) as a seed, the mitochondrial genomes of the *S.spicupennis* and the *S.discalis* were assembled using NOVOPlasty 4.2 (Dierckxsens et al. 2017) and annotated using MITOS Web Server (Bernt et al. 2013). The nucleotide composition of the different regions was analysed with MEGA 7.0 (Kumar et al. 2016). Nucleotide compositional skews were calculated based on the following formulas: AT skew = (A–T)/(A + T) and GC skew = (G–C)/(G + C).

Fourteen specimens were used in the phylogenetic analysis, including the mitogenome newly obtained in this work and 10 other Tetrigidae mitogenomes from GenBank. Mitochondrial genomes of *Mirhipipteryxandensis* and *Ellipesminuta* from Tridactyloidea were selected as outgroups (Table 1). The 13 protein-coding genes were aligned with MAFFT 7.313 (Rozewicki et al. 2019), and the most suitable models for datasets were assessed by ModelFinder (Kalyaanamoorthy et al. 2017). The phylogenetic trees were constructed based on 13 protein-coding genes using the phylogenetic analysis platform Phylosuite (Zhang et al. 2020). Bayesian Inference phylogenies were inferred using MrBayes 3.2.6 (Ronquist et al. 2012) under partition model (2 parallel runs, 2000000 generations), in which the initial 25% of sampled data were discarded as burn-in. Maximum likelihood phylogenies were inferred using IQ-TREE (Nguyen et al. 2015) under Edge-linked partition model for 5000 bootstraps.

**Table 1.** Species and Genbank accession numbers used in this study. Those in bold were generated in this work.

Superfamily / Subfamily /Tribe	Genus	Species	GenBank accession no.
Scelimeninae	*Zhengitettix*	*Zhengitettixcurvispinus*	MT162544
Scelimeninae	*Falconius*	*Falconiuslongicornis*	MT162543
Scelimenini (Scelimeninae)	*Scelimena*	*Scelimenamelli*	MW722938
		*Scelimenadiscalis*	**OP057410** (this study)
		*Scelimenaspicupennis*	**OR333957**
Discotettigini (Scelimeninae)	*Paragavialidium*	*Paragavialidiumsichuanense*	MT162549
		*Paragavialidiumhainanense*	OP650112
Criotettigini (without subfamily placement)	*Criotettix*	*Criotettixjaponicus*	MT162542
Thoradontini (without subfamily placement)	*Eucriotettix*	*Eucriotettixoculatus*	MT162546
	*Loxilobus*	*Loxilobusprominenoculus*	MT162545
	*Thoradonta*	*Thoradontanodulosa*	MT162547
		*Thoradontayunnana*	OP650113
Tridactyloidea	*Mirhipipteryx*	*Mirhipipteryxandensis*	KM657340
	*Ellipes*	*Ellipesminuta*	GU945502

## ﻿Taxonomic account

**Family Tetrigidae Rambur, 1838**

**Subfamily Scelimeninae Bolívar, 1887**

### ﻿Genus Scelimena

Taxon classificationAnimaliaOrthopteraTetrigidae

Serville, 1838

3531BB2A-90A9-5B2E-B33F-6BE4CB88B9AC

Scelimena Serville, 1838: 762; Bolívar 1887: 215; Kirby 1890: 593; Brunner von Wattenwyl and Fea 1893: 103; Hancock 1907: 23; Kirby 1910: 12; Kirby 1914: 21; Hancock 1915: 64; Günther 1938b: 374; Kevan 1966: 377; Shishodia 1991: 16; Blackith 1992: 161; Yin et al. 1996: 908; Otte 1997: 88; Jiang and Zheng 1998: 282; Liang and Zheng 1998: 50; Zheng 2005: 54; Mahmood, et al. 2007: 1278; Deng et al. 2007: 46; Storozhenko and Dawwrueng 2015: 543; Deng 2016: 52; Zha et al. 2017: 372–382; Tumbrinck 2018: 39; Muhammad et al. 2018: 6; Lao et al. 2022: 321; Skejo et al. 2022: 9.

#### Links.

http://orthoptera.speciesfile.org/Common/basic/Taxa.aspx?TaxonNameID=1101762.

#### Type species.

*Scelimenaproducta* Serville, 1838, by monotypy.

#### Description and differential diagnosis.

Members of the genus *Scelimena* have the following characteristics: hind tibiae and the first segment of hind tarsi strongly lamellate; head not exserted; vertex generally wider than or equal to width of the compound eye, a little oblique or inclined anteriorly, extend up to the eyes in front, slightly depressed anteriorly; lateral carinulae lower to the eyes, inclined anteriorly and reflexed laterally, front margin sub-transverse and lower; frontal costa bifurcate between the paired ocelli, narrowly sulcate between paired ocelli and a little widely forked between antennae; paired ocelli placed below the middle rather than on the inferior margin of eyes. Antennae filiform and located below the inferior margin of eyes, Eyes globular and elevated above the vertex. Pronotum transverse anteriorly, subulate posteriorly and extend beyond the apices of hind femora; dorsum rugose, granulose, small tubercles present on the anterior margin below the eyes and also in between, generally distinct tubercles present on the shoulders and lateral margins, sometimes raised linear callosities present on pronotal process; paranota with two projections; posterior angles of lateral lobes of pronotum with a strong spine projecting outwards with its apex more or less directed forwards. Elytra elongate, punctate, apex narrowly rounded. Wings extend up to the apex of pronotum; fore and middle femora elongate, lobate with serrulate margins; posterior femora elongate, crassate, inferior margin frequently dentate; pulvilli of the first segment of hind tarsi more or less equal in length.

*Scelimena* Serville, 1838 is morphologically similar to the genera *Euscelimena* Günther, 1938b, *Indoscelimena* Günther, 1938b and *Paragavialidium* Zheng, 1994, as well as to the genera *Paramphibotettix* Günther, 1938b and *Tagaloscelimena* Günther, 1938b. *Euscelimena*, *Indoscelimena*, *Paramphibotettix* and *Tagaloscelimena* are Scelimenini, while *Paragavialidium* is Discotettigini. In *Euscelimena*, lack of FL2, dentate lower margin of the fore and middle femora. while in *Scelimena*, FL2 present on the anterior margin of pronotum below the eyes, the fore and middle femora lobate with serrulate margins. In *Indoscelimena*, the first segment of hind tarsi is more or less expanded but not lamellated; tubercles weakly developed on the anterior margin below the eyes and in front of the median carina of pronotum, but these are absent on humeral angles and lateral carinae of pronotum. In *Scelimena*, the first segment of hind tarsi with wide lamellar expansions; small tubercles present on the anterior margin of pronotum below the eyes, in front of the median carina of pronotum, on humeral angles and sometimes on the lateral carinae of pronotum also. In *Paragavialidium*, FM forms a distinct projection and the humeral angles of the pronotum project outwards. In *Scelimena*, FM is unrecognizable (except in *S.spicupennis*), the humeral angles of the pronotum not project outwards. In *Paramphibotettix*, the first segment of hind tarsi is more or less expanded but not lamellated. while in *Scelimena*, the first segment of hind tarsi with wide lamellar expansions. In *Tagaloscelimena*, the first segment of hind tarsi with slightly wide lamellar expansions; narrow vertex, lack of FL2. In *Scelimena*, the first segment of hind tarsi strongly lamellate, vertex generally wider than or equal to width of the compound eye, FL2 present on the anterior margin of pronotum below the eyes.

#### Species composition.

There are 23 species distributed in the tropics and subtropics of India, Bangladesh, Myanmar, Thailand, Laos, Vietnam, China, Peninsular Malaysia, Sumatra, Borneo, Palawan Isl., Philippines, Java, East Sumba, Sulawesi, Flores, and New Guinea with adjacent small islands (Muhammad et al. 2018; Cigliano et al. 2022).

### ﻿A key to *Scelimena* species from China

**Table:** 

1	Vertex visibly wider than an eye. Shoulders with ML tubercles	**2**
–	Vertex as wide as an eye or narrower. Shoulders smooth, without ML tubercles	**4**
2	Pronotum smooth, covered in dense granules and without protuberances and notches; lateral carina of pronotum with 1 to 3 pairs of projections (ML1, ML2, ML3), only in the humeral region	***S.pyrroma* Lao, Kasalo, Gao, Deng & Skejo, 2022**
–	Pronotum coarse, its surface with protuberances and concavities; lateral carina of pronotum bearing a row of denticles, with more than 8 pairs of tubercles	**3**
3	Humeral angle arched; projections of pronotal disc indistinct behind the shoulders; teeth of margins of fore and mid femora indistinct	***S.discalis* (Hancock, 1915)**
–	Humeral angles distinctly obtusely angled; pronotum with two pairs of large humps behind the shoulders; teeth of margins of fore and mid femora distinct and large	***S.songkrana* Zha & Wen, 2017**
4	In dorsal view, pronotum with weakly triangular anterior margin; ventrolateral spine (VL) red	***S.spicupennis* Zheng & Ou, 2003**
–	In dorsal view, pronotum with truncated anterior margin; VL not red	**5**
5	FM forming a triangular projection in lateral view; VL directed sidewards	***S.guangxiensis* Zheng & Jiang, 1994**
–	FM unrecognizable in lateral view; VL directed forwards	**6**
6	Vertex as wide as a compound eye; in lateral view, the prozona of the pronotum very undulated; interhumeral carinae recognizable; pronotum dark brown with many yellow dots	***S.melli* Günther, 1938a**
–	Vertex visibly narrower than a compound eye; in lateral view, the prozona of the pronotum weakly undulated, almost flat; interhumeral carinae almost absent; pronotum dark brown	***S.nitidogranulosa* Günther, 1938b**

### ﻿ Scelimena discalis

Taxon classificationAnimaliaOrthopteraTetrigidae

(Hancock, 1915)

41187AD6-2240-547A-AAD7-0F10B8D932BB

Figs 1 , 2 , 3

Eugavialidium discalis Hancock, 1915: 71; Fletcher, 1921: 7.
Scelimena discalis (Hancock, 1915): Günther 1938b: 374; Steinmann 1970: 222; Shishodia 1991: 18; Blackith 1992: 162; Storozhenko and Dawwrueng 2015: 546; Zha et al. 2017: 374; Muhammad et al. 2018: 44.

#### Links.

http://orthoptera.speciesfile.org/Common/basic/Taxa.aspx?TaxonNameID=1101763.

#### Material examined.

P. R. China: Guangxi: 4♂4♀, Duan, 18 August 2015, in CLSGNU; 1♂3♀, Huanjiang (Mulun), 24 September 2016, in CLSGNU.

**Figure 1.**
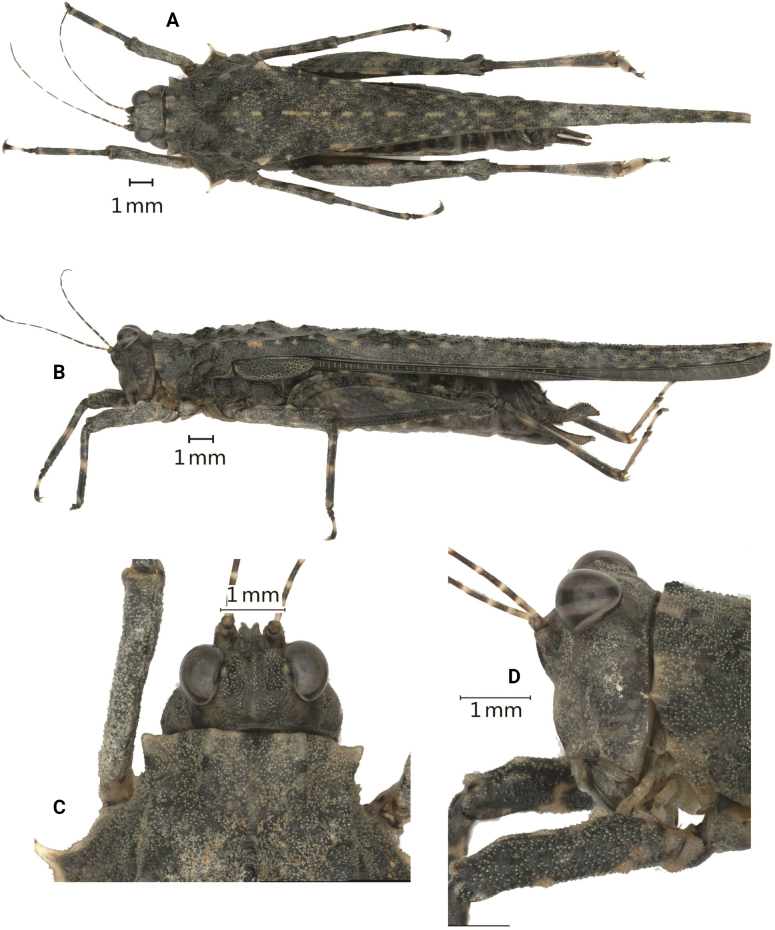
*Scelimenadiscalis* (Hancock, 1915), female **A** body, dorsal view **B** body, lateral view **C** head and anterior part of pronotum, dorsal view **D** head and anterior part of pronotum, lateral view.

#### Redescription.

**Female.** Body large-sized for the genus. Body surface interspersed with coarse protuberances and notches.

**Figure 2.**
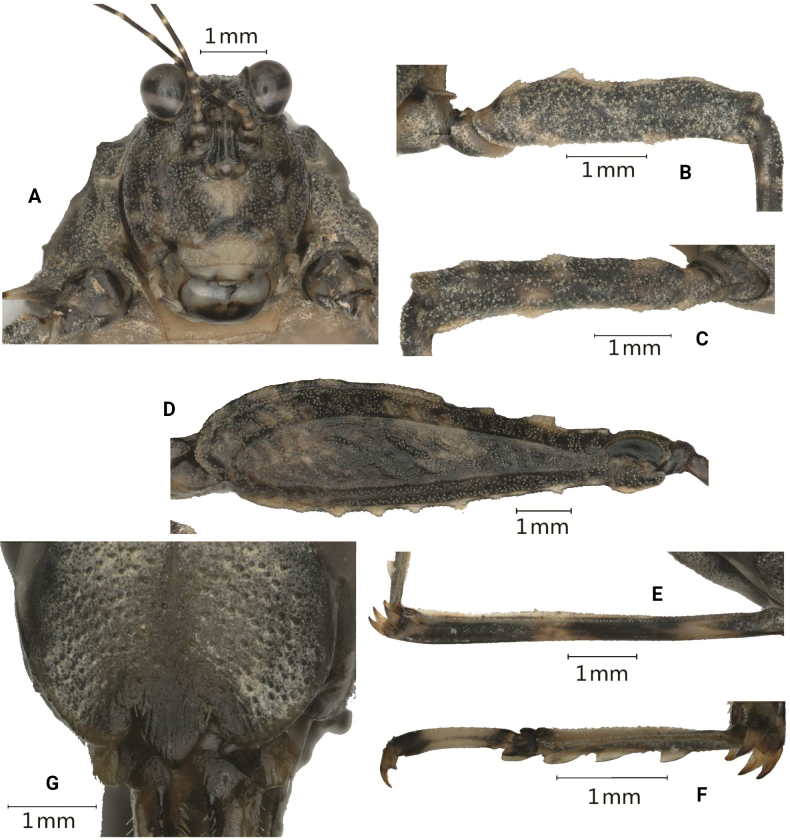
*Scelimenadiscalis* (Hancock, 1915), female **A** head, frontal view **B** right fore femur, lateral view **C** right mid femur, lateral view **D** right hind femur, lateral view **E** right hind tibia, lateral view **F** right posterior tarsus, lateral view **G** subgenital plate of female, ventral view.

***Head*.** Head not exserted above the pronotal surface. The fastigium of the vertex short; in dorsal view, the width of the vertex between eyes 1.4–1.5× the width of a compound eye; the anterior margin of the fastigium nearly straight, not surpassing the anterior margin of the eye; median carina visible anteriorly; lateral margins slightly elevated; vertex uneven with paired fossulae. In lateral view, the frontal costa straight and invisible before the eyes, protruded anteriorly and broadly rounded between the antennal grooves. In the frontal view, the vertex with U-shaped concavity; the frontal costa bifurcated above lateral ocelli, the longitudinal furrow divergent between antennae, width of the longitudinal furrow of the frontal ridge much narrower than the antennal groove diameter. Antennae long, filiform, antennal grooves inserted far below inferior margins of compound eyes, 15-segmented 14-segmented: 1^st^ large scapus, 2^nd^ stout pedicel, 3^rd^ – 6^th^ elongated basal segments, 7^th^ and 8^th^ very elongated mid segments (~ 10.0–12.0× longer than its width), 9^th^ – 11^th^ long subapical segments, 12^th^ – 14^th^ reduced apical segments. Eyes globose, slightly exserted above the pronotal surface in lateral view. Lateral (paired) ocelli located in between inferior margins of compound eye height.

**Figure 3.**
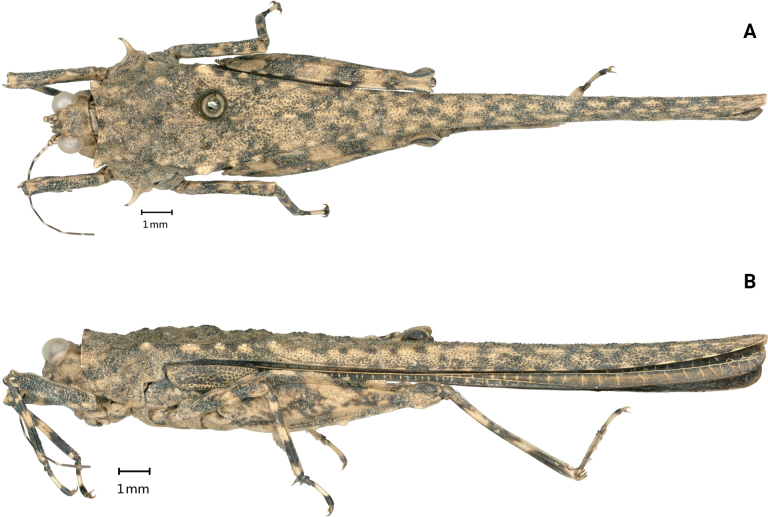
*Scelimenadiscalis* (Hancock, 1915), male **A** body in dorsal view **B** body in lateral view.

***Thorax*.** Pronotum coarse, its surface granulose and with sporadic protuberances and concavities. The pronotum very long (macropronotal state), surpassing the apex of the hind tibiae. Disc of the pronotum gently depressed behind the prozona, slightly swollen between the humeral angles and depressed behind shoulders; bear low and short lineate tubercles between the humeral angles; the middle and posterior parts with sparse short carinae and protuberances and notches. In dorsal view, anterior margin of the pronotum truncated; extralateral projections anteriorly armed with strongly projected FL2, which are pale yellow; FM small, FL1 unrecognizable; anterior half of pronotal median carina entire and posterior half obscure; median carina depressed in front, swollen between sulci and behind the shoulders; lateral carinae in the prozona parallel; humeral angle arched, abbreviated carinae absent; external lateral carinae of pronotum behind the shoulders bear a series of yellowish white denticles (approximately eight or nine): ML strong tubercle directed outward, yellowish white, not spine-like; with a tubercle before ML, but smaller than ML; weak ML2 and ML3 on the external lateral carina behind ML. In profile, the anterior half of the median carina of the pronotum undulated and the posterior half straight; FM minute, PM minute and present as a weak and blunt elevation, MM1 distinct and blunt elevation. MML1 and MML2 small and recognizable, present as small elevations on the dorsum. The ventral margin of the lateral lobe of the pronotum finely serrated, curved forwards, VL strongly produced and sharp. Posterior margins of lateral lobes of pronotum with ventral sinus and tegminal (upper) sinus. Tegmina elongate, punctate, acuminate. Hind wings extend upto the apex of the hind pronotal process.

***Legs*.** Fore and mid femora elongated and not compressed laterally; margins finely serrated, with carinated, with one or two indistinct teeth and slightly undulate; fore femora and mid femora equal in width, width of middle femora distinct narrower than the width of visible part of tegmina. Hind femora elongated, 3.4× as long as wide, dorsal margin finely serrated before the middle and after the middle with three distinct large teeth; antegenicular small and obtuse, genicular denticles small and acute; ventral margin with six or seven teeth. The hind tibia is slightly enlarged from the proximal to distal part, with margins finely serrated, outer side with one or two small spines, and the inner side with four small spines. Fore segment of the hind tarsus widened its width 1.1× the width of the mid femur. Length of the first segment of posterior tarsus longer than third, third pulvillus longer than first and second, apices of first and second acute, apices of third obtuse.

***Abdomen*.** Ovipositor narrow and long, length of upper valvulae 4.5× its width, upper and lower valvulae with slender saw-like teeth. Length of subgenital plate longer than its width, posterior margin of subgenital plate with three teeth.

***Coloration*.** Body dark brown or grey or greyish ferruginous. Antenna black, the area between segments pale. Nodules along the median carina of the pronotum are pale yellow. Tubercles of the external lateral carinae of the pronotum behind the shoulders and VL yellowish white. Fore and mid femora dark brown. Fore and middle tibiae are black, with a pale ring in the middle. The hind femur is dark brown with two or three yellow spots. The hind tibia is black, with two pale rings in the middle.

**Male.** Similar to females, but smaller and narrower. The width of the vertex between the eyes 1.3–1.5× the width of the compound eye; the outer side and the inner side of the hind tibia with 0–1 spine. Subgenital plate short, cone-shaped, apex bifurcated.

#### Measurements (mm).

Length of body: ♂ 11.5–12.5, ♀ 18.5–19.0; length of pronotum: ♂ 23.0–24.5, ♀ 26.5–27.8; length of hind femur: ♂ 7.5–8.0, ♀ 9.0–9.7.

#### Distribution.

Northeast India (Assam), Thailand, and China (Fig. 7). New record from China. May be also present in Myanmar, Laos, and Vietnam.

#### Notes.

*Scelimenadiscalis* is morphologically similar to *S.gombakensis* Muhammad, Tan & Skejo, 2018 from which it differs in dorsal and ventral margins of fore and mid femora with one or two teeth (dorsal and ventral margins of fore and mid femora without tooth in *S.gombakensis*); dorsal margin of hind femora after the middle with three distinct large teeth, ventral margin with six or seven teeth (dorsal and ventral margins of hind femora smooth in *S.gombakensis*).

*Scelimenadiscalis* is similar to *S.songkrana* Zha & Wen, 2017. It differs in that the humeral angle is arched (humeral angles distinctly obtusely angled in *S.songkrana*); projections of pronotal disc indistinct behind the shoulders (pronotum with two pairs of large humps behind the shoulders in *S.songkrana*); teeth of margins of fore and mid femora indistinct (teeth of margins of fore and mid femora distinct and large in *S.songkrana*).

*Scelimenadiscalis* is very similar to *S.chinensis* (Hancock, 1915) from Vietnam, which may be a synonym of *S.discalis*. There is a chance ‘*S.chinensis*’ will be found to be the Chinese ‘*S.discalis*’ in the future, but before the holotype of *S.chinensis* is examined, we retain the name ‘*S.chinensis*’ for this population.

### ﻿ Scelimena spicupennis

Taxon classificationAnimaliaOrthopteraTetrigidae

Zheng & Ou, 2003

DAFF164B-2F26-5D2E-9405-65DC075A5A3D

Figs 4 , 5 , 6

Scelimena spicupennis Zheng & Ou, 2003: 673; Zheng 2005: 55; Deng et al. 2007: 48; Muhammad et al. 2018: 54; Lao et al. 2022: 325.

#### Links.

http://orthoptera.speciesfile.org/Common/basic/Taxa.aspx?TaxonNameID=1101786.

#### Material examined.

4♂5♀, China, Yunnan prov., Mengla (Bubang), 21.628269 N, 101.612976 E, 710 m alt., 27–29 August 2022, collected by Lei Xin, Xiaodong Li and Linyuan Lu, CLSGNU.

**Figure 4.**
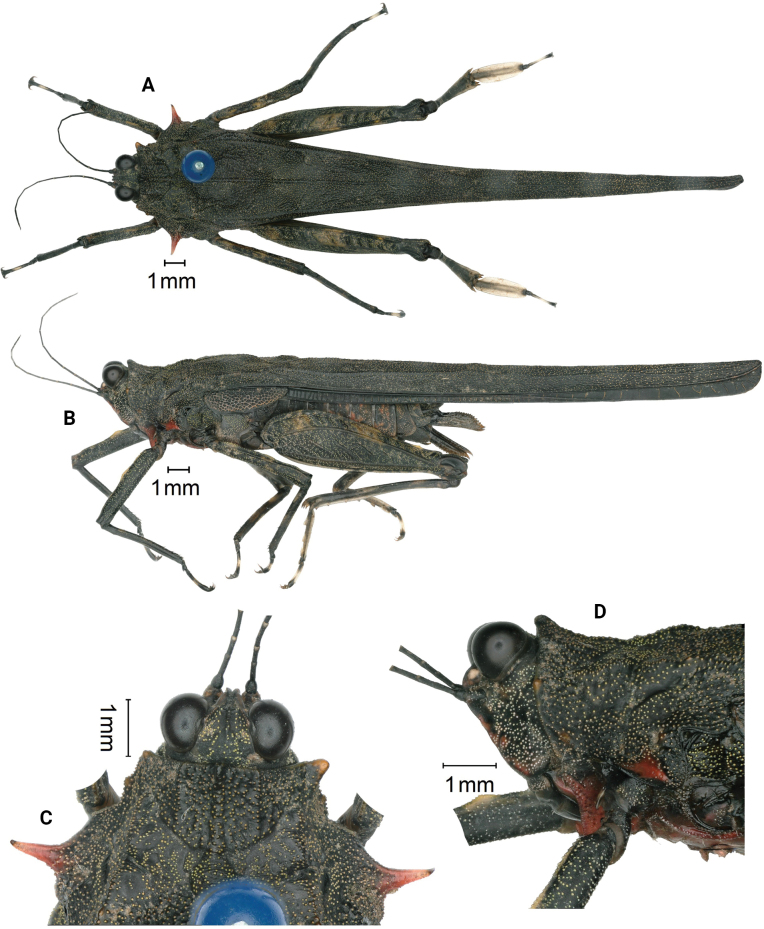
*Scelimenaspicupennis*, female **A** body, dorsal view **B** the same, lateral view **C** head and anterior part of pronotum, dorsal view **D** head and anterior part of pronotum, lateral view.

#### Diagnosis.

This species can be easily distinguished from other species of the genus by the dorsum of pronotum being all dark brown or black, and VL being red. It is morphologically similar to *Scelimenabellula* Storozhenko & Dawwrueng, 2015 from which it differs in FM forming a cylindrical projection in profile (FM unrecognizable in *S.bellula*); dorsum of pronotum is all dark brown or black (external lateral carinae of pronotum are yellow in *S.bellula*); dorsal margin of fore femora after the middle with one indistinct tooth and undulate (dorsal margin of fore femora smooth and straight in *S.bellula*); dorsal margin of hind femora with one large projection before antegenicular denticle (dorsal margin of hind femora smooth in *S.bellula*); sternites of thorax yellow, and sternites of abdomen reddish brown (sternites of thorax and abdomen are reddish brown in *S.bellula*). It is also similar to *S.guangxiensis* Zheng, 1993 but differs from the latter by the pronotum with triangular anterior margin in dorsal view (the pronotum with truncated anterior margin in dorsal view in *S.guangxiensis*); VL red (VL not red in *S.guangxiensis*); lateral spines of lateral lobes of pronotum directed forwards (lateral spines of lateral lobes of pronotum directed sidewards in *S.guangxiensis*); the dorsum of pronotum being all dark brown or black (median carina and discus of pronotum with many yellow dots in *S.guangxiensis*).

**Figure 5.**
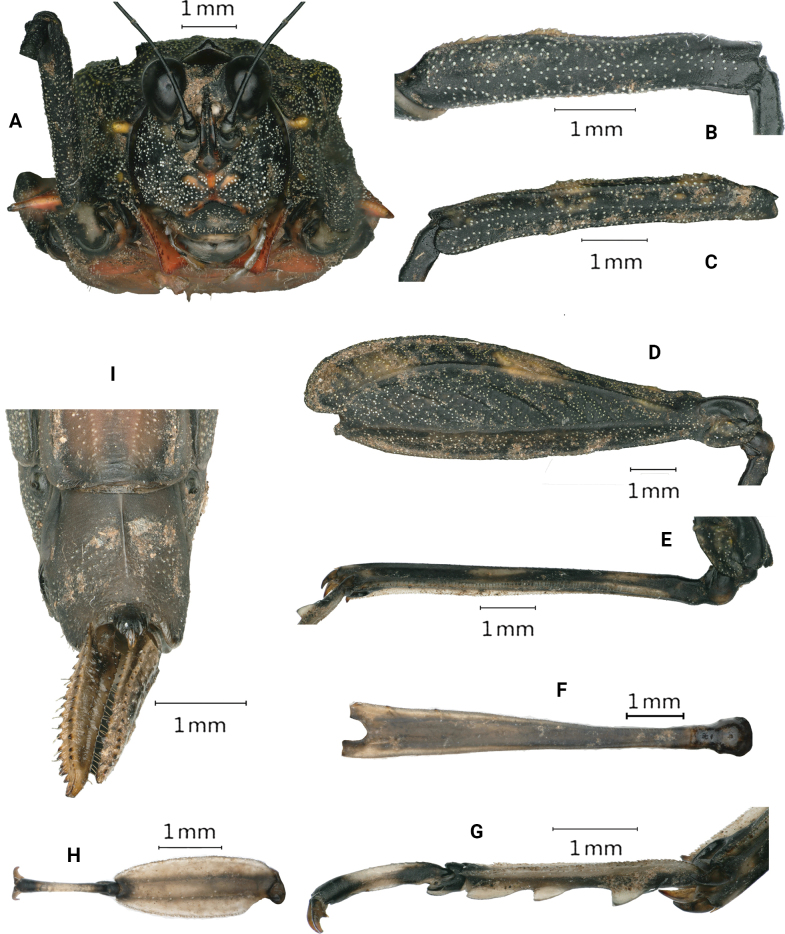
*Scelimenaspicupennis*, female **A** head, frontal view **B** right fore femur, lateral view **C** right mid femur, lateral view **D** right hind femur, lateral view **E** right hind tibia, lateral view **F** right hind tibia, dorsal view **G** right posterior tarsus, lateral view **H** right posterior tarsus, dorsal view **I** subgenital plate of female, ventral view.

#### Redescription.

**Female.** Body large-sized for the genus. Body surface smooth. ***Head*.** Head not exserted above the pronotal surface. Fastigium of vertex short; in dorsal view, the width of the vertex between eyes is nearly equal to the width of compound eye (~ 0.9–1.1×); anterior margin of fastigium narrowly arcuate, not surpassing anterior margin of eye; median carina visible anteriorly; lateral margins turned backward; vertex uneven with paired fossulae. In lateral view, the frontal costa invisible before the eyes, protruded anteriorly and broadly rounded between antennal grooves. In the frontal view, the vertex with V-shaped concavity; the frontal costa bifurcated above lateral ocelli, the longitudinal furrow divergent between antennae, width of the longitudinal furrow of the frontal ridge very narrower than the antennal groove diameter. Antennae long, filiform, antennal grooves inserted slightly below inferior margins of compound eyes (upper margin of the antennal groove at level of inferior margin of the compound eye), 15-segmented, 14-segmented: 1^st^ large scapus, 2^nd^ stout pedicel, 3^rd^ – 6^th^ elongated basal segments, 7^th^ and 8^th^ very elongated mid segments (~ 10.0–12.0× longer than its width), 9^th^ – 11^th^ long subapical segments, 12^th^ – 14^th^ reduced apical segments. Eyes globose, slightly exserted above pronotal surface in lateral view. Lateral (paired) ocelli located slightly below the middle of compound eye height.

**Figure 6.**
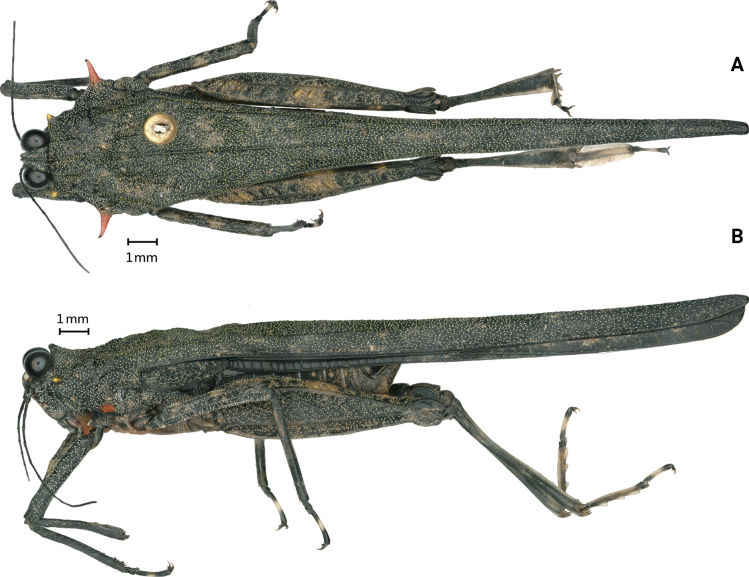
*Scelimenaspicupennis*, male **A** body in dorsal view **B** body in lateral view.

***Thorax*.** Pronotum smooth, its dorsum and external lateral carinae without projection (except for FM, FL2 and an obscure tubercle before the shoulder). Pronotum very long (macropronotal state), surpassing much the apex of the hind tibiae. Disc of the pronotum is gently depressed between the prozona and behind the shoulders, the rest is flat. In dorsal view, pronotum with weakly triangular anterior margin; extralateral projections anteriorly armed with strongly projected FL2, which are yellow; FM small and recognizable, FL1 unrecognizable; median carina of the pronotum continuous from the anterior margin to the tip; lateral carinae in the prozona parallel and indistinct; humeral angle obtuse, abbreviated carinae absent; ML absent, external lateral carinae of metazona behind the shoulders smooth and without denticle. In profile, the anterior half of the median carina of pronotum undulated and the posterior half straight; FM small and cylindrical. The ventral margin of the lateral lobe of pronotum curved forwards, VL is strongly produced and sharp. Posterior margins of lateral lobes of pronotum with ventral sinus and tegminal (upper) sinus. Tegmina elongate, punctate, acuminate; visible part of the tegmen 3.0× as long as wide. Hind wings extend up to the apex of the hind pronotal process.

***Legs*.** Fore and mid femora elongated and not compressed laterally; margins finely serrated, fore femora and mid femora equal in width; dorsal margin of fore femora after the middle with one indistinct tooth and undulate, ventral margin of fore femora straight; dorsal and ventral margins of mid femora slightly undulate, width of middle femora distinctly narrower than the width of the visible part of tegmen. Hind femora elongated, 4.0× as long as wide, dorsal margin and ventral margin finely serrated, dorsal margin with one inconspicuous projection before antegenicular denticle; antegenicular and genicular denticles small and obtuse. Hind tibia strongly widened at the tip, the margins smooth, and outer side and inner side without a spine. The fore segment of the hind tarsus widened its width 1.2× the width of the mid femur, dorsolaterally flattened and forming a swimming paddle. Length of the first segment of posterior tarsus longer than third, third pulvillus longer than first and second, apices of first and second acute, apices of third obtuse.

***Abdomen*.** Ovipositor narrow and long, length of upper valvulae 4.7× its width, upper and lower valvulae with slender saw-like teeth. Length of subgenital plate longer than its width, posterior margin of subgenital plate with three teeth.

***Coloration*.** Body dark brown or black. Antenna black, the area between segments pale. The dorsum of pronotum all dark brown or black; FL2 yellow; VL red. Fore femora and tibiae black; mid femora black, with two yellow spots. Hind femora black, outer dorsal side with two yellow spots. Hind tibia black. The first segment of the posterior tarsus brown. Sternites of thorax yellow, and sternites of abdomen reddish brown; subgenital plate brown.

**Male.** Similar to females, but smaller and narrower. Subgenital plate is short, cone-shaped, apex bifurcated, subgenital plate reddish brown.

#### Measurements (mm).

Length of body: ♂ 13.5–14.0, ♀ 19.5–20.0; length of pronotum: ♂ 24.0–25.0, ♀ 30.5–31.6; length of hind femur: ♂ 8.5–9.0, ♀ 9.5–10.5.

#### Distribution.

**Figure 7.**
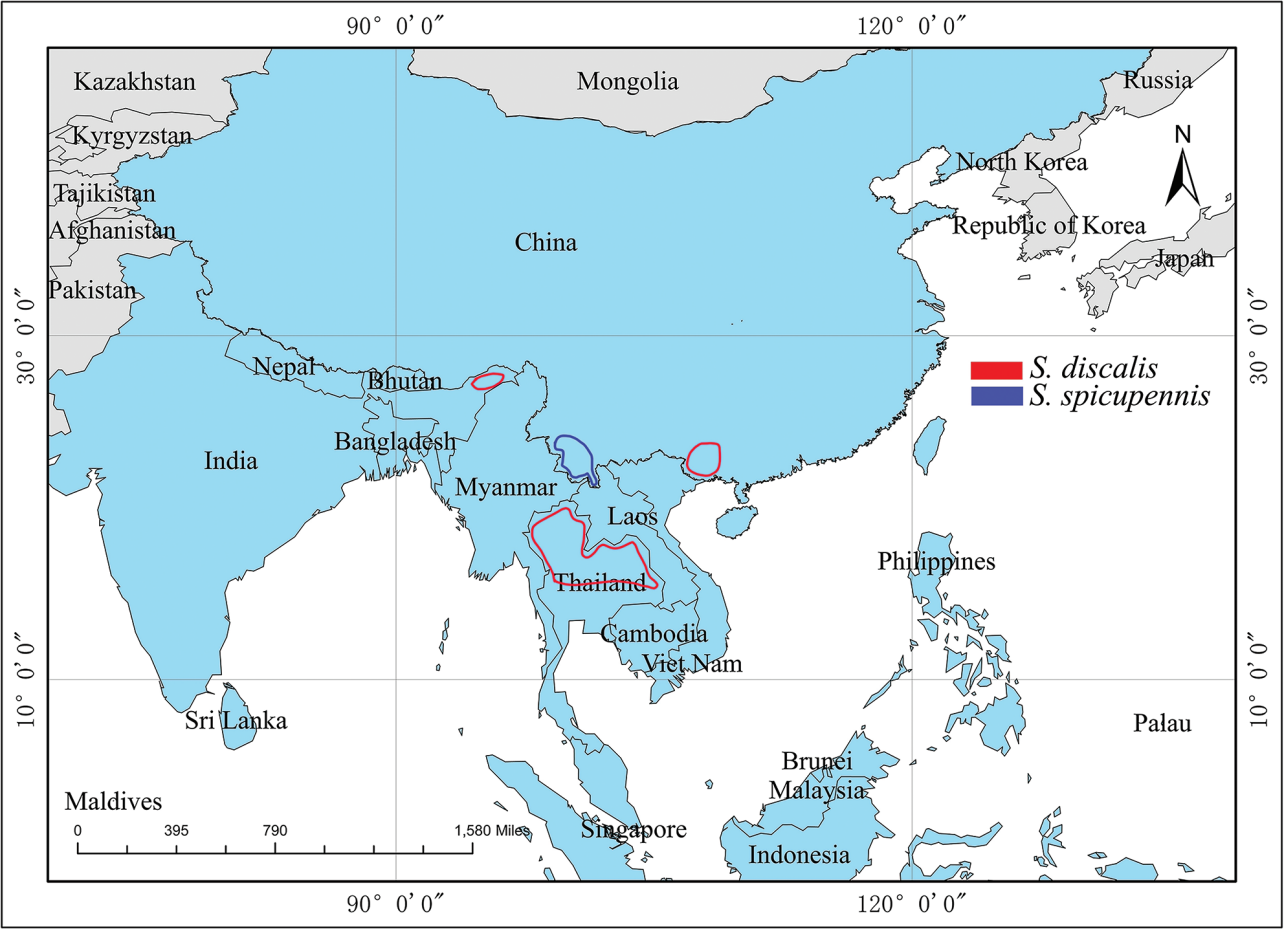
Distribution map of *S.discalis* and *S.spicupennis*.

P. R. CHINA: Yunnan (Fig. 7).

#### Notes.

*Scelimenaspicupennis* is herewith assigned to the *Scelimenabellula* species group due to the dorsum of pronotum being very smooth without recognizable projections, similar to *S.bellula* and *S.guangxiensis*. Metalateral tubercles are absent. It is easily separated from other species of *Scelimenabellula* species group by larger FM and a large depression behind the shoulders of the pronotal disk.

##### ﻿Mitochondrial genomes

###### Analysis of mitochondrial genomes

The sizes of the two sequenced mitogenomes were 17,552 bp (*Scelimenadiscalis*) and 16,069 bp (*Scelimenaspicupennis*). Two mitogenomes had the same gene arrangement and contained 13 protein-coding genes, 22 transfer RNA genes, two ribosomal RNA unit genes, and a noncoding region (A + T-rich regions). The sequence length, direction and codons of each gene in the mitochondrial genomes of the two *Scelimena* species are shown in Tables 2, 3. The arrangement of 37 genes of the two *Scelimena* species was the same as in other Tetrigoidea species (Li et al. 2021); among them, 23 genes (9 protein-coding genes and 14 transfer RNA genes) were located on the majority strand (J-strand), while the remaining genes (four protein-coding genes, eight transfer RNA genes and two ribosomal RNA genes) were encoded on the minority strand (N-strand). The total lengths of the intergenic spacers of the two mitogenomes were 38 bp (*S.discalis*) and 64 bp (*S.spicupennis*). The longest intergenic spacer of *S.discalis* was located between tRNA^Ser2^ and ND1, while that of *S.spicupennis* was located between rrnL and tRNA^Val^. The total lengths of the gene overlaps of the two mitogenomes were 68 bp (*S.discalis*) and 54 bp (*S.spicupennis*). The longest overlapping region of *S.discalis* was between tRNA^Leu1^ and rrnL, and that of *S.spicupennis* was between tRNA^Trp^ and tRNA^Cys^.

**Table 2.** Organization of the *Scelimenaspicupennis* mitogenome.

Name	Strand	Anticodon	Start	Stop	Size(bp)	Ovl(-)/nc(+)	Codons
tRNA^Ile^	J	GAT	1	66	66	0	
tRNA^Gln^	N	TTG	67	135	69	-1	
tRNA^Met^	J	CAT	135	201	67	1	
ND2	J		203	1216	1014	-2	ATC/TAA
tRNA^Trp^	J	TCA	1215	1280	66	-8	
tRNA^Cys^	N	GCA	1273	1335	63	-1	
tRNA^Tyr^	N	GTA	1335	1402	68	-3	
COI	J		1400	2938	1539	-5	ATC/TAA
tRNA^Leu2^	J	TAA	2934	2997	64	0	
COII	J		2998	3678	681	0	ATG/TAA
tRNA^Asp^	J	GTC	3679	3740	62	1	
tRNA^Lys^	J	CTT	3742	3810	69	1	
ATP8	J		3812	3970	159	-7	ATG/TAA
ATP6	J		3964	4635	672	-1	ATG/TAA
COIII	J		4635	5423	789	-1	ATG/TAA
tRNA^Gly^	J	TCC	5423	5485	63	0	
ND3	J		5486	5839	354	-2	ATT/TAG
tRNA^Ala^	J	TGC	5838	5900	63	-1	
tRNA^Arg^	J	TCG	5900	5962	63	0	
tRNA^Asn^	J	GTT	5963	6029	67	0	
tRNA^Ser1^	J	GCT	6030	6096	67	0	
tRNA^Glu^	J	TTC	6097	6159	63	-2	
tRNA^Phe^	N	GAA	6158	6219	62	0	
ND5	N		6220	7945	1726	1	ATG/T(AA)
tRNA^His^	N	GTG	7947	8008	62	-1	
ND4	N		8008	9333	1326	-7	ATG/TAG
ND4L	N		9327	9608	282	8	ATG/TAA
tRNA^Thr^	J	TGT	9617	9680	64	0	
tRNA^Pro^	N	TGG	9681	9743	63	1	
ND6	J		9745	10245	501	-1	TTG/TAA
CytB	J		10245	11384	1140	-2	ATG/TAG
tRNA^Ser2^	J	TGA	11383	11447	65	13	
ND1	N		11461	12402	942	0	ATT/TAA
tRNA^Leu1^	N	TAG	12403	12467	65	-6	
rrnL	N		12462	13734	1273	38	
tRNA^Val^	N	TAC	13773	13839	67	-3	
rrnS	N		13837	14574	738	0	
CR	-		14575	16069	1495		

**Table 3.** Organization of the *Scelimenadiscalis* mitogenome.

Name	Strand	Anticodon	Start	Stop	Size(bp)	Ovl(-)/nc(+)	Codons
tRNA^Ile^	J	GAT	1	66	66	2	
tRNA^Gln^	N	TTG	69	137	69	7	
tRNA^Met^	J	CAT	145	212	68	0	
ND2	J		213	1226	1014	-2	ATT/TAA
tRNA^Trp^	J	TCA	1225	1289	65	-8	
tRNA^Cys^	N	GCA	1282	1344	63	0	
tRNA^Tyr^	N	GTA	1345	1411	67	-3	
COI	J		1409	2947	1539	-5	ATC/TAA
tRNA^Leu2^	J	TAA	2943	3007	65	1	
COII	J		3009	3683	675	4	ATG/TAG
tRNA^Asp^	J	GTC	3688	3748	61	1	
tRNA^Lys^	J	CTT	3750	3817	68	2	
ATP8	J		3820	3978	159	-7	ATG/TAA
ATP6	J		3972	4643	672	-1	ATG/TAA
COIII	J		4643	5431	789	-1	ATG/TAG
tRNA^Gly^	J	TCC	5431	5497	67	0	
ND3	J		5498	5851	354	-2	ATT/TAG
tRNA^Ala^	J	TGC	5850	5913	64	-1	
tRNA^Arg^	J	TCG	5913	5973	61	0	
tRNA^Asn^	J	GTT	5974	6039	66	0	
tRNA^Ser1^	J	GCT	6040	6105	66	0	
tRNA^Glu^	J	TTC	6106	6169	64	-2	
tRNA^Phe^	N	GAA	6168	6229	62	0	
ND5	N		6230	7955	1726	1	ATT/T(AA)
tRNA^His^	N	GTG	7957	8021	65	-1	
ND4	N		8021	9346	1326	-7	ATG/TAG
ND4L	N		9340	9624	285	4	ATG/TAA
tRNA^Thr^	J	TGT	9629	9692	64	0	
tRNA^Pro^	N	TGG	9693	9757	65	1	
ND6	J		9759	10262	504	-1	TTG/TAA
CytB	J		10262	11401	1140	-2	ATG/TAG
tRNA^Ser2^	J	TGA	11400	11464	65	12	
ND1	N		11477	12418	942	0	ATT/TAA
tRNA^Leu1^	N	TAG	12419	12482	64	-23	
rrnL	N		12460	13774	1315	3	
tRNA^Val^	N	TAC	13778	13845	68	-2	
rrnS	N		13844	14584	741	0	
CR	-		14585	17552	2968		

The total length of 13 protein-coding genes of the two mitogenomes was the same (11,125 bp) in *S.discalis* and *S.spicupennis* (Tables 2, 3). For 13 PCGs, the length of each gene ranged from 159 to 1,726 bp, with ATP8 being the shortest (159 bp) and ND5 being the longest (1,726 bp) in both of mitogenomes. All of the PCGs started with the typical ATN (ATT, ATC or ATG) or TTG codon and ended with the complete TAA or TAG codon, with the exception of the ND5 gene, which terminated with an incomplete T. The total lengths of the 22 tRNA genes of the two mitogenomes were 1,433 bp and 1,428 bp. The sizes of the 22 tRNA genes of the two mitogenomes ranged from 61 bp to 69 bp in *S.discalis*, and from 62 bp to 69 bp in *S.spicupennis*. The two ribosomal RNA unit genes, rrnL and rrnS, were located between tRNA^Leu1^ and tRNA^Val^, and between the noncoding region and tRNA^Val^, respectively. The size of rrnL was 1,315bp (*S.discalis*) and 1,273bp (*S.spicupennis*), and the size of rrnS was 741 bp (*S.discalis*)and 738 bp (*S.spicupennis*). The lengths of the noncoding control region were 2,968 bp (*S.discalis*) and 1,495 bp (*S.spicupennis*).

###### Nucleotide composition analysis

The nucleotide characteristics of the newly obtained mitochondrial genome sequence are shown in Tables 4, 5. The nucleotide composition of the two newly sequenced mitogenomes was consistently biased towards A and T nucleotides, which constituted 70.73% (*S.discalis*) and 69.21% (*S.spicupennis*). Comparative analysis showed that the A + T content of the control region was higher than in other regions in *S.discalis* and *S.spicupennis*. The two mitogenomes showed a positive AT skew and a negative GC skew, which indicated that the A and C nucleotides were more abundant than the T and G nucleotides. For 13 PCGs, the overall A + T content was also higher than the G + C content. Moreover, the AT skews were negative, –0.112 (*S.discalis*) and –0.111 (*S.spicupennis*), and the GC skews were slightly negative, –0.026 (*S.discalis*) and –0.056 (*S.spicupennis*). The A + T contents of 22 tRNA genes of the two mitogenomes were 72.23% (*S.discalis*) and 71.79% (*S.spicupennis*) and showed positive AT and GC skews. The A + T content was 73.78% to 72.30% in rRNA genes and showed a negative AT skew and a positive GC skew.

**Table 4.** Nucleotide composition of *Scelimenaspicupennis*.

	T%	C%	A%	G%	A+T%	AT-Skew	GC-Skew
Total	27.64	19.47	41.57	11.32	69.21	0.201	-0.265
PCGs	37.06	17.58	29.65	15.72	66.70	- 0.111	-0.056
tRNA	35.34	12.22	36.45	15.99	71.79	0.016	0.134
rRNA	45.15	9.05	27.15	18.65	72.30	-0.249	0.346
CR	31.97	11.97	48.49	7.56	80.47	0.205	-0.226

**Table 5.** Nucleotide composition of *Scelimenadiscalis*.

	T%	C%	A%	G%	A+T%	AT-Skew	GC-Skew
Total	27.75	19.13	42.98	10.15	70.73	0.215	-0.307
PCGs	37.62	16.59	30.05	15.74	67.67	-0.112	-0.026
tRNA	35.80	12.00	36.43	15.77	72.23	0.009	0.136
rRNA	46.94	8.41	26.85	17.80	73.78	-0.272	0.358
CR	30.76	14.49	48.28	6.47	79.043	0.222	-0.383

Note: AT skew = (A – T) / (A + T); GC skew = (G – C) / (G + C).

###### Phylogenetic analysis

**Figure 8.**
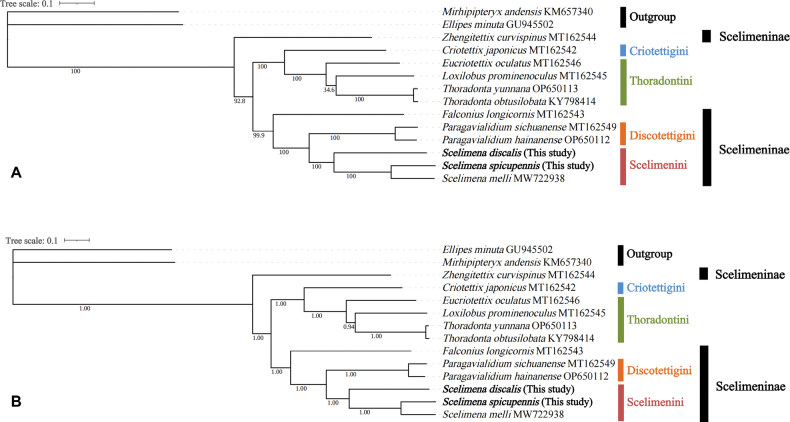
Phylogenetic trees obtained from **A**BI and **B**ML analyses based on 13 protein-coding genes (ND2, COI, COII, ATP8, ATP6, COIII, ND3, ND5, ND4, ND4L, ND6, CytB, ND1).

Using the mitochondrial genomes of the *Mirhipipteryxandensis* and *Ellipesminuta* of Tridactyloidea as outgroups, phylogenetic trees were constructed using BI and ML methods based on the sequences of 13 protein-coding genes from the complete mitochondrial genomes of 12 species of Tetrigidae. The result of the phylogenetic trees, as presented in Fig. 8, indicated that the relationships of the eight genera were as follows: (*Zhengitettix* + ((*Falconius* + (*Paragavialidium* + *Scelimena*)) + (*Criotettix* + (*Eucriotettix* + (*Loxilobus* + *Thoradonta*))))). The three *Scelimena* were clustered into one monophyletic and a holophyletic clade, indicating that they are phylogenetically close and share a common ancestor (Fig. 8). Scelimenini (*Scelimena*) and Discotettigini (*Paragavialidium*) were reconstructed as sister groups.

## Supplementary Material

XML Treatment for Scelimena

XML Treatment for Scelimena discalis

XML Treatment for Scelimena spicupennis

## ﻿Additional information

### Author ORCIDs

Ying-Can Qin https://orcid.org/0009-0000-3859-8261

Jing Liu https://orcid.org/0000-0002-8477-6572

Xiao-Dong Li https://orcid.org/0000-0002-0443-7715

Ya-Zhen Chen https://orcid.org/0000-0003-0900-9020

Wei-An Deng https://orcid.org/0000-0002-8023-2498

### Data availability

All of the data that support the findings of this study are available in the main text.
